# Athletes’ self-compassion and emotional resilience to failure: the mediating role of vagal reactivity

**DOI:** 10.3389/fpsyg.2023.1192265

**Published:** 2023-06-09

**Authors:** Nan Zhang, Jiasheng Huang, Jiaxin Yao

**Affiliations:** ^1^School of Psychology, Beijing Sports University, Beijing, China; ^2^Department of Psychology, Sun Yat-sen University, Guangzhou, China; ^3^College of Education and Psychology, Tianjin University of Sport, Tianjin, China

**Keywords:** self-compassion, emotional resilience, failure, vagal reactivity, mediation

## Abstract

Whether athletes’ self-compassion predicts their emotional resilience to failure has yet to be empirically tested. Moreover, as an important physiological process of stress regulation, vagal reactivity is a plausible physiological mechanism for this relationship. Through a laboratory-based observational study of 90 college athletes, this research explores the influence of athletes’ trait self-compassion on their emotional resilience when recalling failure, and examines whether vagal reactivity plays a mediating role. The results show that self-compassion did not significantly predict athletes’ positive emotions but did significantly predict better recovery from negative emotions after recalling failure events. Furthermore, vagal reactivity was a significant mediator between self-compassion and recovery from negative emotions.

## 1. Introduction

In sports competitions, all athletes expect to succeed, but there is only one champion, so failure is an inevitable and frequent outcome for most participants. When an athlete fails, especially in an important game, possible consequences include depression, anger, and mental fatigue (Jones and Sheffield, 2007; Hammond et al., 2013), leading to a decline in performance. The emotional devastation of failure may even make some athletes end their sporting careers prematurely. Thus, it is important to investigate how athletes can develop emotional resilience to failure, which refers to generating positive emotions and recovering quickly from the negative emotional experiences caused by failure (Davidson, 2000).

Previous studies have shown that athletes tend to engage in severe self-criticism, self-judgment, and rumination when encountering failures (Mosewich et al., 2013; Ferguson et al., 2014). This is especially typical of Chinese athletes: they compete not only for themselves but also for their team and country. Should they fail, the voice of self-criticism and self-condemnation becomes stronger (Wang and Shi, 2012). Many athletes believe this way of relating to themselves is necessary for success in elite sports, and without it, they will become complacent and fail to reach their potential (Sutherland et al., 2014; Rodriguez and Ebbeck, 2015). However, the outcomes can be counterproductive. Research showed that self-criticism or self-punishment undermined athletes’ self-regulation, emotional recovery, stress management, and performance (Powers et al., 2009; Tenenbaum et al., 2013; Ferguson et al., 2015). These harsh attitudes were also positively associated with negative emotional responses, avoidance, and fear of failure (Sagar et al., 2007; Powers et al., 2009).

By contrast, treating oneself with compassion may be a more beneficial way to respond to failure. Self-compassion entails three main components: (a) self-kindness—being kind and understanding toward oneself in instances of pain or failure, rather than being harshly self-critical; (b) common humanity—perceiving one’s experiences as part of the larger human experience, rather than seeing them as separating and isolating; and (c) mindfulness—holding painful thoughts and feelings in balanced awareness, rather than over-identifying with them (Neff, 2003). When facing difficulties, people with self-compassion have the desire to soothe themselves rather than ruminate and overidentify painful experiences and have motivation and action to pursue happiness (Neff, 2003; Allen and Leary, 2010; Barnard and Curry, 2011; Terry and Leary, 2011). Self-compassion also has particular benefits for athletes, helping them to develop adaptive thoughts, emotions, and behavioral responses to stress (Mosewich et al., 2013; Ferguson et al., 2015; Reis et al., 2015; Ceccarelli et al., 2019), as well as realizing their potential under adversities (Ferguson et al., 2014; Lizmore et al., 2017). Moreover, evidence in the general population suggests that self-compassion may facilitate emotional resilience to negative events. For example, studies have found that people with high self-compassion tend to take a more balanced approach to their negative experiences—neither avoiding nor dwelling on negative emotions—and recover more quickly (Neff and Vonk, 2009; Poots and Cassidy, 2020). Mantelou and Karakasidou (2017) also found that changes in self-compassion on the part of the intervention group could predict changes in positive affect. That means that participants who face everyday challenging situations in a more self-compassionate manner are also to gain more in terms of positive effect. However, compared with the general population, athletes face tremendous pressure over a long period; they are used to being strict with themselves and particularly worry that self-compassion could lead to complacency, mediocrity, or passivity (Ferguson et al., 2014). Thus, the contributive role of self-compassion to athletes’ emotional resilience to failure remains questionable and has yet to be explored.

Vagal reactivity may be an important physiological mechanism through which self-compassion promotes better emotional resilience failure in athletes. The vagus nerve is a component of the parasympathetic branch of the autonomic nervous system (Beauchaine, 2001). It helps individuals maintain homeostasis in response to changes in the external environment by regulating organs. The Polyvagal theory posits that vagus nerve activity is an important neurophysiological basis of emotion and social behavior (Porges, 1995, 2001, 2003, 2007). When the external environment is relatively safe, the parasympathetic system plays a leading role in physiological regulation, with the vagus nerve strongly contributing to maintaining the homeostasis of the internal environment and directing appropriate emotional and behavioral responses. By contrast, when the external environment changes and the individual is in a state of stress, the sympathetic nerve plays a leading role, and the regulation of the vagus nerve on the heart will be weakened, resulting in vagal withdrawal (Porges, 2001). Vagal reactivity is the strength of responsiveness of the vagus nerve during such a withdrawal process. High vagal reactivity indicates individuals’ flexibility in coping with challenges caused by environmental changes (Porges et al., 1996; Porges, 2007; Santucci et al., 2008).

In stressful situations such as failure, higher vagal reactivity may help athletes generate adaptive emotional responses, thereby facilitating their emotional resilience. Prior studies have revealed a positive association between vagal reactivity and appropriate emotional responses in other populations. For example, using discussion of anger events to induce negative emotions in participants, Cui et al. (2015) found that higher vagal reactivity was associated with better regulation of anger and sadness and more prosocial behavior in adolescents induced with negative emotions. Obradović et al. (2010) showed that children with high vagal reactivity showed more positive emotions toward social activities under low adversity conditions. Moreover, Calkins and Keane (2004) found that children with higher vagal reactivity throughout preschool years had fewer negative emotions, behavioral problems, and better social skills than those with lower vagal reactivity.

Self-compassion, as a healthy attitude toward oneself, may promote better vagal reactivity in individuals. Gilbert (2009) suggests that caring and supportive self-talk has similar effects to early attachment experiences. On this basis, self-compassion is linked to feelings of safety because one knows that failure or mistakes will not result in severe self-condemnation. According to Porges’ polyvagal theory, when individuals feel safe, they tend to have higher physiological flexibility (Thayer and Lane, 2000; Porges, 2001). Thus, as an indicator of physiological flexibility, vagal reactivity should be improved by better self-compassion. Researchers have also explored the relationship between self-compassion and vagal reactivity to acute stress events, finding that self-compassion predicted athletes’ vagal reactivity upon stress induction (Ceccarelli et al., 2019). A 12-week compassion intervention study also showed that improving self-compassion could enhance vagal reactivity in a distressed clinical sample (Steffen et al., 2021). However, no studies have explored the relationship between self-compassion and vagal reactivity in Chinese athletes. Based on the evidence above, it is plausible that self-compassion contributes to greater emotional resilience to failure in athletes, possibly through physiologically enhanced vagal reactivity.

### 1.1. Present research

**Figure 1:**
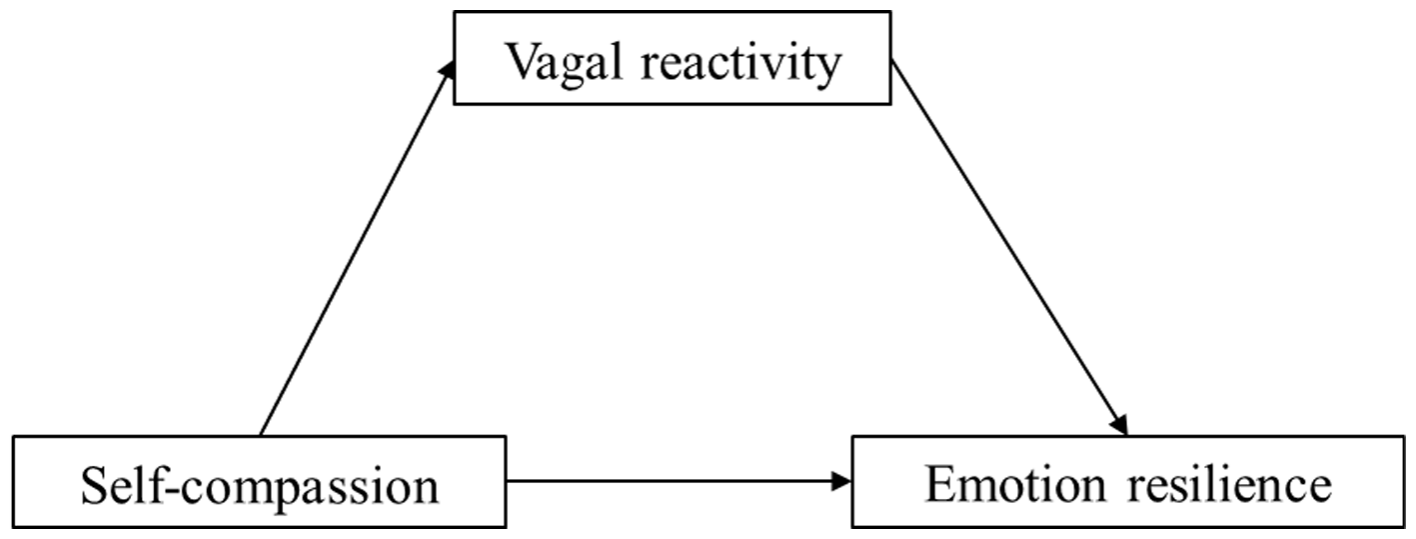
The hypothesized mediation model.

In summary, self-compassion may be important for athletes to maintain emotional resilience in the face of failure, but this potential relationship needs to be empirically tested. As suggested by the polyvagal theory, self-compassion may influence emotional resilience to failure through vagal reactivity. Thus, it is important to investigate whether vagal reactivity, as a fundamental physiological process of stress regulation, is the underlying mechanism of this relationship. Accordingly, we conducted a laboratory-based observational study in a sample of college athletes to explore the influence of trait self-compassion on emotional resilience to failure and to verify whether vagal reactivity plays a mediating role (Figure 1). We specifically tested four hypotheses:

*Hypothesis 1*: Self-compassion predicts athletes’ better emotional resilience to failure (i.e., more positive emotions and better recovery from negative emotions after recalling failure events).

*Hypothesis 2:* Self-compassion predicts higher vagal reactivity to recalled failure events in athletes.

*Hypothesis 3*: Athletes’ vagal reactivity predicts better emotional resilience to failure.

*Hypothesis 4*: Vagal reactivity plays a mediating role between athletes’ self-compassion and emotional resilience to failure.

## 2. Methods

### 2.1. Participants

Using G*Power to calculate the sample size, with *f^2^* = 0.15, *α* = 0.05, and *power* = 0.80, we determined that at least 89 participants were required. We recruited participants from a sports university in Shandong Province, China through online advertisements. Participants were required to: (1) have experience in official competition; (2) not have physical conditions that might interfere with the measurement of HRV (e.g., concussion, sleep deprivation, and severe illness; Svendsen et al., 2016; Laborde et al., 2017); and (3) have the experience of sporting failures that they could clearly recall. 101 eligible participants were recruited, but six did not complete baseline measures, and five were unavailable for the laboratory session. Demographic information of the final sample is presented in Table 1. The sample comprised 90 college athletes (60 men, 30 women; mean age 20.79 ± 1.18 years; age range: 18–23 years). Participants competed in various fields, with handball (13.3%) and gymnastics (11.1%) most strongly represented.

**Table 1:** Demographic information of participants.

	*N* (%) / *M* ± *SD*
Gender
Male	60 (66.7)
Female	30 (33.3)
*Age*	20.79 ± 1.19
Training experience
< 5 years	33 (36.7)
5–10 years	54 (60.0)
11–15 years	3 (3.3)
Sporting level
International athlete	1 (1.1)
National athlete	2 (2.2)
National first-level athlete	41 (45.6)
National second-level athlete	6 (6.7)
Other	40 (44.4)
Highest competition experience
National level	33 (36.7)
Provincial level	20 (22.2)
City level	20 (22.2)
Other	17 (18.9)

### 2.2. Procedures

Two days before the experiment, participants were informed not to consume any substances that may affect the stress response (such as alcohol, sedatives, and sleep aids) and to maintain a good sleep pattern (Svendsen et al., 2016; Laborde et al., 2017), aiming to ensure that their physiological data were as reliable as possible. The experiment was conducted in a laboratory without being interrupted. After giving fully informed consent, participants reported demographic information and completed the self-compassion measure. Next, the researcher helped each participant wear the physiological measurement equipment and instructed them to sit comfortably and relaxed with both of their feet flat on the floor, hands on their thighs, and palms facing up (Laborde et al., 2017) for 2 min (T1). Participants were told to remain still, as movement could interfere with the measurements. Next, physiological baseline data were recorded over a 5-min resting period (T2), with participants given the same instructions as during T1 and then asked participants to recall past sport failures (Ceccarelli et al., 2019) for 2 min (T3), again following the same instructions as those given at T1. Throughout the 2 min, the researcher read aloud cues prompting participants to recall their past failures. The prompts were adapted from the research of Ceccarelli et al. (2019). Each prompt was followed by a brief pause to give participants time to visualize the recalled failures. The prompts were as follows:

Please recall a sport failure that happened in the past…Maybe you made a costly mistake, failed to meet an important goal, or experienced a setback in your sport progress?…Really try to take yourself back to this experience…What happened then?…Who was there?…And what about the surrounding environment?… How did you feel when this happened?… Maybe disappointment, anger, frustration, hopelessness?… Try to recall these feelings in as much detail as possible… Allow yourself to feel them… Recall the feelings of your body… tension, anxiousness, uneasiness… Imagine the scene in as much detail as possible…Try to bring yourself back to the feelings and emotions you are experiencing, as if back in that moment of failure… Now, please take a deep breath and gently open your eyes.

The recalling period (T3) was followed by a 5-min recovery period (T4), in which participants were asked to close their eyes but given no other prompts. Their emotional state was measured immediately after T3 and at the end of every minute during T4, with six measurements in total. Finally, participants rated the emotional difficulty and visual clarity of the event.

### 2.3. Measures

#### 2.3.1. Demographics

Each participant reported age, gender, grade, specialist sport, training experience, sporting level, and highest competition experience.

#### 2.3.2. Self-compassion

Participants completed the Self-Compassion Scale compiled by Neff (2003), comprising 26 items across six subscales that assess the three dimensions of self-compassion and their opposites: mindfulness (overidentification), self-kindness (self-criticism), and universal humanity (isolation). All items are scored on a five-point scale, ranging from 1 (*almost never*) to 5 (*almost always*). Higher scores on this scale indicate higher levels of self-compassion. In this study, the Self-Compassion Scale had good reliability (α = 0.87).

#### 2.3.3. Emotional resilience

It is suggested that emotional resilience can be indicated by individuals’ ability to generate positive emotions and recover from negative emotions after adverse events (Davidson, 2000). Therefore, this study used the mean levels and the changes of positive and negative emotions after recalling failure events to measure athletes’ emotional resilience to failure. Positive and negative emotional states are measured on different visual analog scales (Wolpe, 1990). For positive emotional state, 0 represents very calm, and 100 represents the most positive/pleasant emotional state, while for negative emotional state, 0 represents very calm, and 100 represents the most negative/unpleasant emotional state. Two indices were generated to assess positive emotion: the mean positive emotion and the rate of change in positive emotion during the recovery period (T4). Mean positive emotion was the average of the six repeated measures of positive emotion throughout T4. Meanwhile, the rate of change in positive emotion is measured by the slope obtained by regressing measurement time points (1–6) on the rated scores of positive emotion throughout T4. The larger the value of these two indicators, the better emotional resilience in terms of positive emotions. Mean negative emotion and the rate of change in negative emotion during the recovery period were generated in the same way. The smaller the value of the two indicators, the better emotional resilience in terms of recovery from negative emotions.

#### 2.3.4. Vagal reactivity

Participants’ HRV was measured using a Polar H10 chest strap (Rockliff et al., 2008; Arch et al., 2014). This device collects and processes HRV measurements by detecting electrical signals from the heart. The device was connected to the Elite HRV app on an iPad via Bluetooth 4.0. After data collection, the original R-R interval data in Elite HRV were downloaded to a Windows 10 laptop as a text file. They were imported to Kubios HRV software (version 3.1.0, Biosignal Analysis and Medical Imaging Group, University of Kupio, Finland, MATLAB).

Vagal reactivity can be reflected by the inhibitory effects of vagus on sinus node, which can be quantified by heart rate variability (Thayer and Sternberg, 2006). Therefore, as many previous studies did, our study used heart rate variability to measure vagal reactivity (Muhtadie et al., 2015; Stellar et al., 2015; Luo et al., 2018; Di Bello et al., 2020). There are two methods for measuring heart rate variability, time-domain and frequency-domain indicators. Studies using these two indicators showed relatively consistent findings regarding the relation between HRV and emotional response, as well as self-compassion (Eisenberg et al., 1996; Geisler et al., 2010; Kirby et al., 2017; Lischke et al., 2019; Di Bello et al., 2020). However, research suggested that while time-domain indicators such as RMSSD could be influenced by sympathetic input, frequency-domain indicators such as HF-HRV might better reflect cardiac vagal activity (Berntson et al., 1993, 2005). Moreover, prior studies also showed that HF-HRV was applicable in measuring the vagal activity of athletes (Davydov et al., 2015; Laborde et al., 2015; Ceccarelli et al., 2019). Therefore, this study used HF-HRV to measure vagal reactivity. Kubios was used to calculate HF-HRV (0.15–0.4 Hz, absolute units). HF-HRV values were log-transformed to better approximate a normal distribution in order to conform to parametric assumptions. In our study, we chose threshold-based correction to correct artifacts and ectopic beats. The number of corrected beats was controlled at a level of less than 5%. Drawing on the approach of Muhtadie et al. (2015), we calculated the baseline HF-HRV using HRV records throughout the last minute of T2, then calculated HF-HRV reactivity by subtracting the baseline HF-HRV (T2) from HF-HRV during the first minute of T3 (Stellar et al., 2015). For ease of interpretation, we multiplied HF-HRV reactivity by −1 so that the more significant number of HF-HRV reactivity denoted greater vagal reactivity (Muhtadie et al., 2015).

#### 2.3.5. Validity of recall

We used two items to assess the validity of recall by reference to previous studies investigating athletes’ responses to hypothetical and recalled scenarios of adverse events (Reis et al., 2015; Ceccarelli et al., 2019). Participants rated the emotional difficulty they experienced when recalling the failure event, using a six-point scale ranging from 1 (not at all) to 6 (extremely). They are also asked to report the visual clarity of the recalled sport failure, using a seven-point scale ranging from 1 (very blurred) to 7 (very clear).

### 2.4. Data analysis

SPSS 27.0 was used in this study to conduct descriptive statistics and regression analysis. Descriptive statistics were used to analyze the scores of the three variables and failure-induced manipulation tests. The macro program PROCESS of SPSS developed by Hayes (2013) was used to examine the mediating role of vagal reactivity between self-compassion and emotional resilience. First, a simple mediation analysis was performed. The analysis of the simple mediation effect model is to implement the following linear regression equation:

(1) $$Y=cX+\varepsilon_{1}$$

(2) $$M=aX+\varepsilon_{2}\;$$

(3) $$Y=c^{\prime }X+bM+\varepsilon_{3}\;$$

where the regression coefficient *c* of equation (1) is the effect of independent variable *X* on dependent variable *Y*; the regression coefficient *a* of equation (2) is the effect of independent variable *X* on mediator variable *M*; the regression coefficient *b* of equation (3) is the effect of *M* on *Y* after controlling the effect of *X*; the coefficient *c*′ is the effect of *X* on *Y* after controlling the effect of *M*; ε_1_, ε_2_, and ε_3_ represent residuals, assuming that the residuals follow a normal distribution and are independent of each other. Substituting (2) into (3), we get

(4) $$Y=\left(c^{\prime }+ab\right)X+\varepsilon_{2}b+\varepsilon_{3}$$

*ab* in equation (4) is the mediation effect of the independent variable *X* on the dependent variable *Y*; *c’* is the direct effect of *X* on *Y*, and *c’* + *ab* is the total effect of *X* on *Y* (total effect), that is *c* = *c’* + *ab*. The mediation effects were tested with the Bootstrap method, in which the sample was repeatedly sampled 5,000 times (Preacher and Hayes, 2008). The confidence intervals (CI) of 95% were generated based on the bootstrapped results, and a 95% CI that did not include 0 indicated a significant mediation effect.

## 3. Results

The scores of emotional difficulty (*M* = 3.86/*6, SD* = 1.09) and visual clarity (*M* = 4.70/7, *SD* = 1.17) of the recalled sport failure were both above the mid-point of the corresponding scales (i.e., 3.5 and 4, respectively). Results indicated that the failure events were generally validly and effectively recalled (Ceccarelli et al., 2019).

The results of mediation analyses are presented in Table 2. Controlling for age and gender, results showed that higher self-compassion significantly predicted both a smaller rate of change in negative emotion (*c* = −0.08, *SE* = 0.03, *p* = 0.01) and lower mean negative emotion (*c* = −0.35, *SE* = 0.15, *p* = 0.03). However, self-compassion failed to significantly predict the rate of change in positive emotion (*c* = 0.01, *SE* = 0.04, *p* = 0.86) and mean positive emotion (*c* = *−*0.04*, SE* = 0.20*, p* = 0.85). Thus, higher self-compassion significantly predicted better recovery from negative emotions, but not more positive emotions, after recalling a sport failure, which partially supported Hypothesis 3.

**Table 2:** Mediation analyses of vagal reactivity between self-compassion and emotional resilience.

Path	*B*	*SE*	*p*	95%CI
SC → VR	0.37	0.17	0.03	[0.035, 0.697]
VR → Negative emotion change rate	−0.06	0.02	0.008	[−0.098, − 0.015]
SC → VR → Negative emotion change rate	0.02	0.01	–	[−0.052, − 0.003]
VR → Mean negative emotion	−0.24	0.10	0.02	[−0.428, − 0.046]
SC → VR → Mean negative emotion	−0.09	0.05	–	[−0.221, − 0.010]
VR → Positive emotion change rate	0.01	0.03	0.66	[−0.070, 0.044]
SC → VR → Positive emotion change rate	0.004	0.01	–	[−0.020, 0.029]
VR → Mean positive emotion	−0.10	0.12	0.45	[−0.350, 0.158]
SC → VR → Mean positive emotion	0.03	0.05	–	[−0.071, 0.152]

SC, self-compassion; VR, vagal reactivity; and CI, confidence intervals.

Table 3 is the HF-HRV values of different periods. As shown in Table 2, higher self-compassion significantly predicted higher vagal reactivity when recalling a sport failure (*a* = 0.37*, SE* = 0.17*, p* = 0.03), thus supporting Hypothesis 2.

**Table 3:** HF-HRV values of different periods.

Variables	*M*	*SD*
T1-2 min HF-HRV	51.87	20.48
T2-1 min baseline HF-HRV	49.56	21.64
T3-1 min recall failures	54.99	20.80

Higher vagal reactivity significantly predicted both smaller rate of change in negative emotion (*b* = −0.06, *SE* = 0.02, *p* = 0.008) and lower mean negative emotion (*b* = −0.24, *SE* = 0.10, *p* = 0.02). However, vagal reactivity significantly predicted neither the rate of change in positive emotion (*b* = 0.01, *SE* = 0.03, *p* = 0.66) nor mean positive emotion (*b* = *−*0.10*, SE* = 0.12*, p* = 0.45). These results showed that higher vagal reactivity significantly predicted better recovery from negative emotions, but not more positive emotions, after recalling a sport failure, which partially supported Hypothesis 3.

Vagal reactivity was found to have significant mediating effects between self-compassion and, respectively, the rate of change in negative emotion (*ab* = 0.02, *SE* = 0.01, 95% CI [−0.052, −0.003]) and mean negative emotion (*ab* = −0.09, *SE* = 0.05, 95% CI [−0.221, −0.010]). These indirect effects are 0.099 and 0.023, respectively, accounting for 25 and 24% of the total effects. Vagal reactivity did not significantly mediate self-compassion and, respectively, the rate of change in positive emotion (*ab* = 0.004, *SE* = 0.012, 95% CI [−0.020, 0.029]) and mean positive emotion (*ab* = 0.03, *SE* = 0.054, 95% CI [−0.071, 0.152]). The results partially supported Hypothesis 4. Self-compassion significantly predicted neither the rate of change in negative emotion (*c’* = 0.06, *SE* = 0.03, *p* = 0.06) nor mean negative emotion (*c’* = *−*0.26*, SE* = 0.15*, p* = 0.09) after controlling for the effects of vagal reactivity.

**Figure 2:**
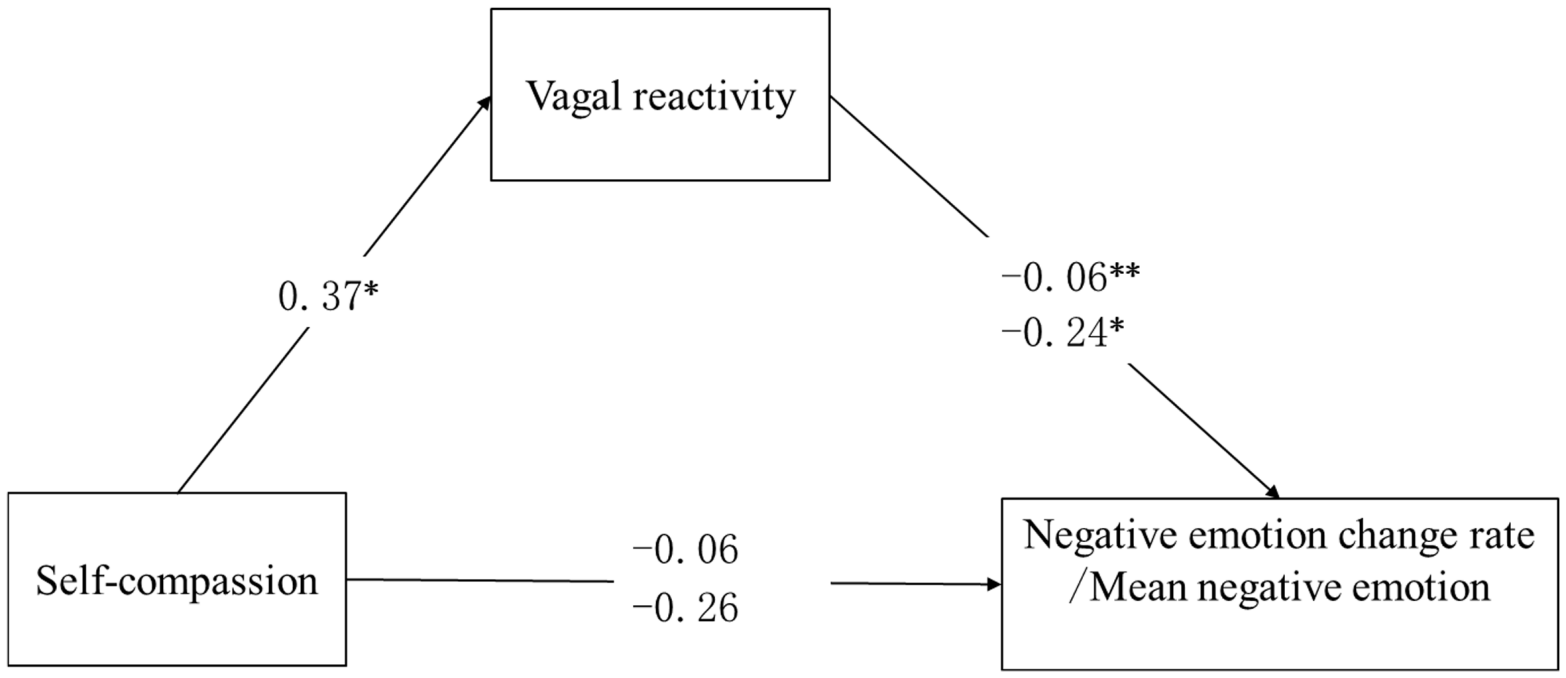
Results of the mediation model. ^*^p < 0.05 and ^**^p < 0.01. The upper coefficient is the standardized regression coefficient when the outcome variable is negative emotion change rate. The lower coefficient is the standardized regression coefficient when the outcome variable is mean negative emotion.

Self-compassion was not significantly associated with rate of change in negative affect (*c*’ = 0.06, SE = 0.03, *p* = 0.06) and mean negative affect (*c*’ = −0.26, SE = 0.15, *p* = 0.09; Figure 2).

## 4. Discussion

The primary objective of this study was to examine the effect of athletes’ self-compassion on their emotional resilience to failure (i.e., positive emotions and recovery from negative emotions), as well as the potential mediating role of vagal reactivity. Results showed that self-compassion did not significantly predict athletes’ positive emotions, but higher self-compassion did significantly predict better recovery from negative emotions after recalling failure events. Furthermore, vagal reactivity when recalling failure events significantly mediates self-compassion and recovery from negative emotions.

### 4.1. Self-compassion and athletes’ emotional resilience to failure

Our results demonstrated that higher self-compassion could significantly predict better emotional resilience in terms of recovery from negative emotions: athletes with higher self-compassion demonstrated less negative emotion and a faster recovery rate of negative emotion after recalling a failure event. This is consistent with previous results in the general population (Neff and Vonk, 2009; Poots and Cassidy, 2020), which have shown that people with high self-compassion tend to engage less in excessive thinking and thought inhibition (Neff, 2003; Neff and Knox, 2016) and typically take a more balanced approach toward their emotional experience, neither avoiding nor dwelling on negative emotions (Neff and Vonk, 2009; Poots and Cassidy, 2020). Consequently, individuals with high self-compassion can recover faster from negative emotions. Athletes are more accustomed to treating themselves harshly than the general population and worry that self-compassion will hinder their progress (Ferguson et al., 2014). However, our findings showed a positive connection between self-compassion and recovery from negative emotions after recalling a failure in an athlete sample. When failures or setbacks occur, it is important for athletes to recover quickly from negative emotions. Because negative emotions could narrow people’s momentary thought–action repertoire (Fredrickson, 2001) and lead to energy depletion (Ekman, 1999; Lundqvist and Kenttä, 2010), which might substantively impair athletes’ performance, therefore failure to rapidly recover after a negative event can increase vulnerability to emotional disorders, particularly for an individual frequently exposed to negative events (Davidson, 2000). So developing emotional resilience can help athletes recover quickly from setbacks in training or competition and, ultimately, positively impact their performance, long-term health and well-being. While failures are inevitable and frequent in athletes’ careers, our findings highlight the possible role of self-compassion in athlete development because this positive psychological trait might help build up athletes’ ability to better recover from negative emotions caused by failure.

However, this study did not find significant associations between athletes’ self-compassion and positive emotional experiences after recalling sport failure. This finding is inconsistent with the results for Western populations (Mantelou and Karakasidou, 2017). However, prior studies have shown that mindfulness interventions could effectively improve positive emotions in Western people (Anderson et al., 2007; Schroevers and Brandsma, 2010) but not in Chinese populations (Liu et al., 2013). Researchers have suggested that Chinese culture may explain this difference, which emphasizes the state of tranquility—contrasting with the hedonism and self-realization view of happiness in Western culture (Gao et al., 2010). Thus, Chinese people may place higher values on safe or content positive affect (e.g., safe) and relaxed positive affect (e.g., calm) than activated positive affect (e.g., excited; Gilbert et al., 2008). Thus, the influence of self-compassion on positive emotional experience after failure may be more manifested as the effect on safe/content/calm affect in the Chinese population. This study used a single item to broadly measure participants’ positive emotional state, and so may not have captured the impact of self-compassion on athletes’ experience in terms of safe/content/calm positive affect. Therefore, future research should specifically measure safe/content/calm positive affect, for instance, by using the Affect Balance Scale or the Activation and Safe/Content Affect Scale (Gilbert et al., 2008).

### 4.2. The mediating role of vagal reactivity

More importantly, we found that vagal reactivity mediated the relationship between self-compassion and athletes’ emotional resilience to failure. To the best of our knowledge, this is the first study exploring the mechanism by which self-compassion affects emotional resilience in athletes. Specifically, our findings showed that athletes with higher self-compassion had higher vagal reactivity when recalling a failure event, thereby demonstrating better recovery from negative emotions after recalling the failure.

Our findings showed that higher self-compassion predicted higher vagal reactivity to failure. This is consistent with previous results in Western groups (Ceccarelli et al., 2019; Steffen et al., 2021). Our study replicates prior findings in a sample of Chinese athletes. This association suggests that self-compassion may benefit athletes’ physiological flexibility in times of stress. According to the Cognitive Activation Theory of Stress (Ursin and Eriksen, 2004), cognitive processes may extend the duration of physiological stress responses. Thus, when an athlete is unable to regulate negative thoughts about the stressful situation, the stress response would not be “switched off,” and the psychophysiological activation would remain high, which can undermine the functioning of physiological systems (Gilbert, 2009). While people with higher self-compassion tend to have a better ability to accept and tolerate negative experiences (Neff et al., 2005; Diedrich et al., 2014) and use less maladaptive regulation strategies such as avoidance, thought suppression, and rumination (Neff et al., 2005, 2007; Barnard and Curry, 2011), they may thereby be equipped with better physiological resources to stress.

While higher self-compassionate athletes possessed higher vagal reactivity in the face of failure, they could thereby recover better from negative emotions after the event. Such findings are consistent with the polyvagal theory, which argues that vagal reactivity is the basis for flexible adaptation (Porges, 1995, 2001, 2003, 2007). On this basis, athletes with higher vagal reactivity are more likely to respond effectively to changes in the environment. When negative emotions occur due to undesirable stimuli such as failure, these athletes may show more adaptive emotional responses and faster recovery from negative emotions. This is crucial in highly competitive sports, in which athletes’ performance is substantively affected by their momentary mental and physical state: rapid and adaptive physiological and emotional responses to incidental failures may be critical determinants of ultimate victory and long-term progress.

The positive role of self-compassion in athletes’ vagal reactivity and emotional resilience to a failure we found in this study further emphasizes the importance of self-compassion for athletes. Previous studies have also shown that self-compassion positively influences athletes’ adaptive thoughts, behaviors, and potential personal development (Ferguson et al., 2014; Lizmore et al., 2017; Ceccarelli et al., 2019). Worth noted, self-compassion has been found intervenable via different types of well-established clinical programs (e.g., Mindful self-compassion program; Neff and Germer, 2013 and compassion-focused therapy; Gilbert, 2009). In the sporting domain, Mosewich et al. (2013) also developed a 7-day self-compassion intervention for athletes. Evidence from a randomized control trial showed that female athletes receiving this intervention demonstrated enhanced self-compassion, as well as decreased self-criticism and rumination, and these improvements were maintained 1 month later. These findings, together with ours, implicate the practical utility of training self-compassion to facilitate athletes’ career development. Future studies may further develop intervention programs of self-compassion and test their efficacy in a broader sample of athletes.

Finally, our study has several limitations. First, this study relied on recalled rather than actual failure stimuli, which may bias athletes’ physiological and emotional responses. Future research could examine the relationship between self-compassion and emotional resilience and the mediating role of vagal reactivity under standardized laboratory stressor conditions or in real-life failure scenarios in training or competition. Second, as mentioned above, our measure of positive emotion did not differentiate the types of positive affect, which may have neglected the culture-specific emphasis on safe/content positive affect or relaxed positive affect in the Chinese population. We recommend future research to add measures of safe/content/calm positive affect to further examine the influences of self-compassion on athletes’ positive feelings of safety or contentment after failure. Third, we assessed vagal reactivity using a 1-min HF-HRV mean. Although measurements over such a short period can be considered reliable (Esco and Flatt, 2014; Laborde et al., 2017), future research should test the robustness of our findings by measuring HF-HRV over a longer time period. Fourth, our findings do not allow inferences of causality because this study did not involve manipulations of self-compassion or vagal reactivity. Future studies could employ appropriate experimental designs to examine the causal relationship between self-compassion and emotional resilience to failure and the mediating role of vagal reactivity.

## Data availability statement

The raw data supporting the conclusions of this article will be made available by the authors, without undue reservation.

## Ethics statement

The studies involving human participants were reviewed and approved by College of psychology, Beijing Sports University. The patients/participants provided their written informed consent to participate in this study.

## Author contributions

NZ, JH, and JY: conceptualization and writing—review and editing. NZ and JH: data curation. NZ: investigation and writing—original draft. All authors contributed to the article and approved the submitted version.

## Funding

This work was supported by the National Natural Science Foundation of China (82103956) and the MOE (Ministry of Education) Project of Humanities and Social Science of China (21YJCZH042).

## Conflict of interest

The authors declare that the research was conducted in the absence of any commercial or financial relationships that could be construed as a potential conflict of interest.

## Publisher’s note

All claims expressed in this article are solely those of the authors and do not necessarily represent those of their affiliated organizations, or those of the publisher, the editors and the reviewers. Any product that may be evaluated in this article, or claim that may be made by its manufacturer, is not guaranteed or endorsed by the publisher.
